# Editorial: Decoding the genetics of viral evolution

**DOI:** 10.3389/fgene.2022.978485

**Published:** 2022-08-11

**Authors:** Hashim Ali, Sheikh Abdul Rahman, Junaid Akhtar, Syed Asfarul Haque, Safdar Ali

**Affiliations:** ^1^ Division of Virology, Department of Pathology, Addenbrooke’s Hospital, University of Cambridge, Cambridge, United Kingdom; ^2^ Division of Microbiology and Immunology, Emory Vaccine Center, Emory National Primate Research Center, Emory University, Atlanta, GA, United States; ^3^ Institute of Developmental Biology and Neurobiology, University of Mainz, Mainz, Germany; ^4^ St. Jude Children’s Research Hospital, Memphis, TN, United States; ^5^ Clinical and Applied Genomics (CAG) Laboratory, Department of Biological Sciences, Aliah University, Kolkata, India

**Keywords:** virus, evolution, genetics, mutation, SARS-CoV-2

There are multiple facets to successfully tackling the menace of viral diseases. These include monitoring the sequence variations and emergence of new viruses/variants which has been the backbone of understanding the COVID-19 pandemic. Furthermore, a clear understanding of the physiological and clinical aspects of viral pathogenesis helps to identify diagnostic and therapeutic targets (Laskar and Ali, 2021a; Laskar and Ali, 2021b; Laskar and Ali, 2021c).

SARS-CoV-2 has been the most widely studied virus in the shortest period of time since its emergence in December 2019 which led to multiple successful vaccination programs. The challenges posed herein extends beyond health aspects and has had both social and economic implications. A few percent fatality rate leads to severe complications in those patients. Zeyaullah et al.

However, neither SARS-CoV-2 nor some of the viruses known for decades like HIV or Hepatitis viruses are fully understood till date. Hepatitis B virus (HBV) is a small DNA virus belonging to the “Hepadnaviridae” family. It infects liver cells and is transmitted through direct contact with infected blood or body fluids. Despite the successful development of the HBV vaccine, still, approx. 300 million people are infected with HBV, with 1 million deaths and over 1.5 million new infections annually (World Health Organization. Global Progress Report on HIV, Viral Hepatitis and Sexually Transmitted Infections. 2021). HBV-positive individuals have a high potential risk of developing liver cirrhosis and hepatocellular carcinoma (HCC) than uninfected ones. However, the precise molecular mechanisms driving HBV-induced HCC have been poorly characterised. During virus-host genome integration and prolonged inflammation (Feitelson and Lee, 2007; Berasain et al., 2009), HBx protein alters the cellular homeostasis and thus plays a decisive role in the progression of the liver tumorigenesis (Ali, 2014).

HBx protein, a small regulatory protein is pivotal in reprogramming both nuclear and cytoplasmic signalling pathways of the HBV-infected cells. Sequencing studies have identified high rates of mutations in the viral genomes recovered from HBV individuals (Kao et al., 2000). It has been shown that the HBxΔ127 mutant favours the proliferation of host cells and is thus potentially involved in HCC development (Wang et al., 2010; World Health Organization, 2021). However, the mechanism of how HBxΔ127 is engaged in HCC development remains unclear. Siddiqui et al. have shown how the expression of a truncated version of HBx can affect molecular and cellular processes and contribute to the development of HBV-related liver diseases.

In terms of SARS-CoV-2, the key aspect for exploration has been interaction of spike protein with human ACE2 receptor. This interaction has been the basis of enhanced infectivity of Omicron variant as shown by multiple studies. Thus, exploration of potential drugs to inhibit this interaction was then logical choice. A study on screening of various drug candidates and exploration revealed anidulafungin and lopinavir to be potent against ACE2 receptor-S glycoprotein interaction. The clinical data to support the same is required for further authentication. Ahamad et al.

The emergence and havoc caused by the beta and omicron variants have highlighted the significance of monitoring of sequences and different examples from across the world suggest that sequencing and contact tracing have been the most effective in restricting the spread of COVID-19. The mutational analysis of SARS-CoV-2 strains from patients flying from Bangladesh to Italy in July 2020 revealed that these strains were not further observed in Italy due to the screening at the point of entry and following restrictive measures. Rueca et al.

Extending this thought is the prediction of viral evolution which has been identified as emergence potential in several motifs and viral sequences of Togaviridae, Arenaviridae, and Flaviviridae families. Similar efforts are required on a constant basis to prepare us for another pandemic. Mazur et al.
